# A Hepatitis C Virus DNA Vaccine Encoding a Secreted, Oligomerized Form of Envelope Proteins Is Highly Immunogenic and Elicits Neutralizing Antibodies in Vaccinated Mice

**DOI:** 10.3389/fimmu.2019.01145

**Published:** 2019-05-24

**Authors:** Makutiro Ghislain Masavuli, Danushka K. Wijesundara, Alexander Underwood, Dale Christiansen, Linda Earnest-Silveira, Rowena Bull, Joseph Torresi, Eric J. Gowans, Branka Grubor-Bauk

**Affiliations:** ^1^Virology Laboratory, Basil Hetzel Institute for Translational Medicine, Discipline of Surgery, University of Adelaide, Adelaide, SA, Australia; ^2^Faculty of Medicine, The Kirby Institute, School of Medical Sciences, University of New South Wales, Sydney, NSW, Australia; ^3^Department of Microbiology and Immunology, The Peter Doherty Institute for Infection and Immunity, University of Melbourne, Parkville, VIC, Australia

**Keywords:** hepatitis C vaccine, viral hepatitis, HCV envelope proteins, preventative vaccination, DNA vaccine, immune response, liver disease, IMX313P

## Abstract

Hepatitis C virus (HCV) persistently infects approximately 71 million people globally. To prevent infection a vaccine which elicits neutralizing antibodies against the virus envelope proteins (E1/E2) which are required for entry into host cells is desirable. DNA vaccines are cost-effective to manufacture globally and despite recent landmark studies highlighting the therapeutic efficacy of DNA vaccines in humans against cervical cancer, DNA vaccines encoding E1/E2 developed thus far are poorly immunogenic. We now report a novel and highly immunogenic DNA vaccination strategy that incorporates secreted E1 and E2 (sE1 and sE2) into oligomers by fusion with the oligomerization domain of the C4b-binding protein, IMX313P. The FDA approved plasmid, pVax, was used to encode sE1, sE2, or sE1E2 with or without IMX313P, and intradermal prime-boost vaccination studies in BALB/c mice showed that vaccines encoding IMX313P were the most effective in eliciting humoral and cell-mediated immunity against the envelope proteins. Further boosting with recombinant E1E2 proteins but not DNA nor virus-like particles (VLPs) expressing E1E2 increased the immunogenicity of the DNA prime-boost regimen. Nevertheless, the antibodies generated by the homologous DNA prime-boost vaccinations more effectively inhibited the binding of VLPs to target cells and neutralized transduction with HCV pseudoparticles (HCVpp) derived from different genotypes including genotypes 1, 2, 3, 4, 5, and 6. This report provides the first evidence that IMX313P can be used as an adjuvant for E1/E2-based DNA vaccines and represents a translatable approach for the development of a HCV DNA vaccine.

## Introduction

Current estimates have shown that more than 71 million people are living with chronic hepatitis C and a large proportion of these individuals are at risk of developing cirrhosis that may progress to hepatocellular carcinoma (1, 2). In 2015, viral hepatitis caused 1.34 million deaths and surpassed human immunodeficiency virus (HIV) as a cause of mortality worldwide (1). The introduction of direct acting antivirals (DAA), developed to disrupt the function of critical non-structural (NS) HCV proteins involved in viral replication, has been highly effective, resulting in a reduced duration of treatment and high cure rates in most patients, compared to pegylated interferon plus ribavirin (3). However, the high cost of the DAA limits their use particularly in low- to middle-income regions (4–6). Furthermore, it is believed that only 20% of HCV-infected persons are aware of their diagnosis while approximately 7.4% (or 1.1 million persons) of those diagnosed receive treatment and an estimated 1.75 million new HCV infections occurred worldwide in 2015 (1). An additional challenge to DAA treatment includes the potential for reinfection after treatment (7). For these reasons, the development of an inexpensive, safe and effective HCV vaccine is required to control the HCV epidemic worldwide and reduce the financial stress on healthcare systems.

The development of a prophylactic HCV vaccine has been difficult due to several factors including the high genetic heterogeneity of the virus and its ability to escape the immune defense of the infected host. The early induction of broad neutralizing antibodies (NAb) and CD4^+^ and CD8^+^ T cell responses directed against HCV structural and NS proteins are thought to correlate with recovery from acute HCV infection (8–11). The HCV envelope glycoproteins (E1 and E2) interact with several receptor proteins, including CD81 and the tight junction proteins claudin (CLDN) and occludin (OCLN) (12, 13) to facilitate virus entry into host cells (14, 15). Consequently, E1 and E2 have been the targets of various trials which aimed to induce protective NAb (16–23). Previous studies have shown that NAb to epitopes in the HCV E1 and E2 are protective against several genotypes of HCV (24–29). Besides NAb, numerous studies have shown that the induction of cell-mediated immunity (CMI) is linked to the clearance of HCV infection (30) and depletion of CD4^+^ or CD8^+^ T cells has been reported to lead to persistent infection in chimpanzees (31, 32). Therefore, a vaccine capable of eliciting NAb and T-cell immunity is desirable.

The vaccine efficacy data from HCV challenge studies in chimpanzees [reviewed in (33)], further highlighted the importance of NAb in providing protection against HCV. Previous reports have demonstrated that NAb present in HCV patient sera are protective against homologous- (34) and heterologous- HCV challenge in passively immunized chimeric human liver SCID/uPa mice (35, 36). Vaccination of these mice with a recombinant vaccinia virus vaccine encoding the HCV structural proteins was also shown to generate protection against HCV challenge (37). Chimpanzees vaccinated with recombinant E1 and E2 proteins produced in mammalian cells were protected against persistent infection after homologous or heterologous HCV challenge (16). Furthermore, cross-reacting, broad NAb against several HCV genotypes were generated following immunization with a recombinant HCV E1E2 vaccine (18, 21).

The removal of the respective transmembrane domains (TMD) of E1 and E2 can result in the secretion of truncated forms of these proteins into the extracellular milieu (38, 39). Vaccination with the secreted form of the E1/E2 antigens led to an increased antibody response in mice (39, 40) as secretion of the antigens increase their chances of capture by antigen presenting cells (APCs), resulting in increased immunogenicity (41–43). However, it is difficult to elicit anti-E1 responses which have been reported to require the separation of the E1E2 heterodimer, suggesting that the E2 protein is immunodominant or that E2 expression masks the immunogenic epitopes on E1 (18). Furthermore, a recent study using a vaccine candidate based on chimeric hepatitis B virus (HBV)-HCV envelope particles, showed that E1- and E2-specific antibody responses were profoundly impaired in animals immunized with VLPs expressing a E1E2 heterodimer compared to animals vaccinated with VLPs expressing E1 and E2 separately (44). Additionally, the E1- and E2-specific antibodies were able to cross-neutralize numerous other HCV genotypes, emphasizing the significance of including E1 and E2 as separate immunogens to induce both E1- and E2-specific antibody response (44).

DNA-based vaccination offers a unique alternative method of immunization. DNA vaccines can induce both CMI and antibody responses, result in persistent expression of heterologous antigen and elicit a memory response toward the antigen (45, 46). Additionally, DNA vaccines are safe in humans, and are easy and cost effective to produce on a large-scale (47). However, DNA vaccines often induce poor immune responses in large animals despite being effective in mice (45), and require adjuvants for maximum effect (46). Co-administration or co-expression of a DNA vaccine with a plasmid encoding immunomodulatory proteins, such as interleukin (IL)-12 (48) or cytolytic adjuvants such as perforin (49–53) represent strategies employed to boost antigen-specific immunity following DNA vaccination.

Oligomerization is used by various proteins to increase protein valency, structural stability and binding affinity (54). Recently, fusion of protein antigens to a chimeric version of the oligomerization domain from the chicken complement inhibitor C4b-binding protein (C4 bp) (termed IMX313), was reported to result in self-assembly into soluble heptameric structures after expression, resulting in improved antibody responses compared to the same dose of monomeric antigen (55–57). Furthermore, mice vaccinated with the malaria vaccine candidate MSP119 fused to IMX313 were protected against challenge with a lethal dose of *Plasmodium yoetii* parasites (55). Other reports have shown that vaccination of mice with the *Mycobacterium tuberculosis* antigen 85A fused to IMX313 in both viral vector and DNA vaccines resulted in consistently increased CD4^+^ and CD8^+^ T cell responses in mice and increased magnitude of the immune response in mice and non-human primates (56). Furthermore, a recent phase I clinical trial of a viral vector encoding 85A-IMX313 in healthy BCG (Bacillus Calmette-Guerin)-previously vaccinated adults revealed that the vaccine was well-tolerated and immunogenic (58) (clinicaltrials.gov ref. NCT01879163). More recently a DNA vaccine, encoding a secreted form of the HIV Tat protein fused to IMX313, elicited anti-Tat NAb, Tat-specific CMI and protection against challenge with a chimeric HIV, EcoHIV, in mice (59).

Therefore, the aim of this study was to examine the immunogenicity of a DNA vaccine encoding secreted HCV E1 and/or E2 after fusion with a modified form of IMX313, namely IMX313P. Since the adjuvanticity of IMX313 or IMX313P requires the protein to be effectively secreted (55–57), a tissue plasminogen activator (tPA) leader sequence was introduced upstream of the truncated E1 or E2 proteins (sE1 or sE2) from which the TMDs were removed. Additionally, because the optimal approach of processing and presenting these proteins for potent immunization is yet to be defined, the efficacy of DNA vaccines encoding sE1 and sE2 proteins as separate immunogens or as a single truncated sE1E2 polyprotein fused to IMX313P was assessed in BALB/c mice after intradermal prime-boost DNA immunizations or after boosting DNA immunized mice with sE1E2 proteins or VLPs expressing E1E2.

## Materials and Methods

### DNA Plasmid Construction

**Figure 1:**
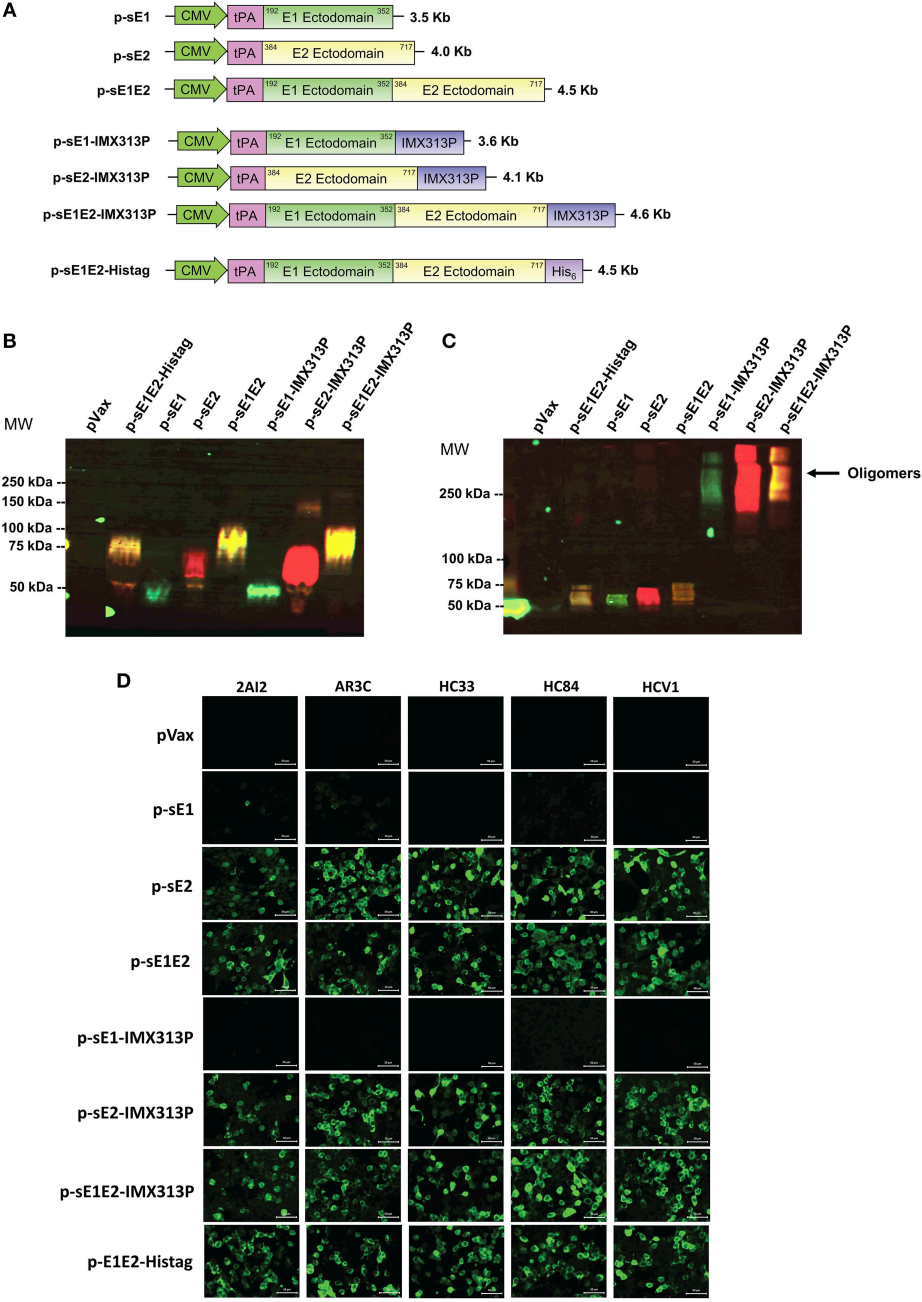
Construction and validation of DNA vaccine constructs. **(A)** Schematic diagram of the DNA constructs used in the study. DNA vaccines encoding sE1, sE2, or sE1E2 were produced by fusion to an N-terminal signal peptide, tPA, including one construct with a C-terminal hexahistidine (His6) tag. Constructs encoding secreted envelope proteins fused to the IMX313P domain were also generated. The numbering coincides with the amino acid positions in the polyprotein of the HCV genotype 1b strain (GenBank accession number AF139594.2). DNA plasmid sizes are shown on the right. Western blot analysis of HCV proteins in concentrated cell culture media harvested from HEK293T cells transfected with DNA constructs encoding secreted envelope with or without IMX313P under **(B)** reducing and **(C)** non-reducing conditions, and probed with mouse anti-E1 or human anti-E2 MAb HCV1 primary antibodies, followed by mouse anti-AF488 (green signal) or human anti-AF555 (red signal). The yellow signal represents the co-detection of E1 and E2. Molecular weight (MW) markers are indicated on the left in kilodaltons and HCV protein oligomers are indicated on the right. pVax-transfected cells represent controls to show non-specific bands. **(D)** Immunofluorescence analysis HEK293T cells transfected with the different DNA vaccines and controls were stained with human MAbs. Scale bar = 50 μm.

Codon-optimized genes (Gene Art, Germany) encoding gt1b HCV E1 and E2 (GenBank accession number AF139594.2) were used in a series of overlapping PCRs to remove the E1 and E2 TMD and introduce the signal peptide sequence of the tissue plasminogen activator (tPA) upstream of E1 or/and E2. These genes were inserted into pVax downstream of the cytomegalovirus (CMV) promoter and the tPA leader sequence to generate p-sE1, p-sE2, or p-sE1E2 (Figure 1A). Similarly, the codon optimized IMX313P gene was introduced downstream of the E1 and/or E2 genes to generate p-sE1-IMX313P, p-sE2-IMX313P, or p-sE1E2-IMX313P. p-sE1E2-Histag plasmid encoding secreted E1/E2 fused to a 6 × His tag was used to express E1 and E2 in HEK293T cells for ELISA. The bicistronic plasmid p-CE1E2-PRF(DA), encoding full length HCV (gt1b) core, E1 and E2 proteins under the control of the CMV promoter and a non-cytolytic version of perforin [PRF(DA)] containing a D483A mutation (50–61) under the control of the simian virus 40 (SV40) promoter, was used to express the native structural proteins in HEK cells as an antigen target in immunofluorescence. DNA constructs used in immunizations were prepared using the Endotoxin Free Plasmid Giga Kit from Qiagen following the manufacturer's instructions.

### Immunofluorescence

Human embryonic kidney 293T (HEK293T) cells were cultured in 96 well plates in Dulbecco modified Eagle's medium (DMEM, Life Technologies) supplemented with 1% penicillin-streptomycin (Life Technologies) and 10% fetal calf serum at 37°C in 5% CO_2_. The cells were transfected with 200 ng of DNA using Lipofectamine LTX (Life Technologies) according to the manufacturer's protocol. Thirty-six hours post-transfection, the cells were fixed in 4% paraformaldehyde then permeabilized with 100% methanol. The cells were then incubated with the appropriate primary antibody at 37°C for 2–4 h at 37°C or overnight at 4°C followed by the addition of the appropriate fluorophore-conjugated secondary antibody for 1 h at 37°C in the dark. Primary antibodies used include mouse anti-E1 (MyBiosource, Cat. No. MBS310203), goat anti-E2 (Virostat, Cat. No. 2851), pooled sera collected from vaccinated mice or human MAb (kindly supplied by Heidi Drummer). Secondary antibodies were Alexa Fluor (AF) 488-conjugated anti-mouse, AF488-conjugated anti-human and AF555-conjugated anti-goat (all from Invitrogen). Nuclei were stained with Hoechst 33342 (Life Technologies). Cells were visualized by fluorescence microscopy (Zeiss LSM-700) and the data digitized using the Zen software (Zeiss).

### Western Blotting Analysis

To confirm E1/E2 expression, supernatant fluids were harvested from HEK293T cells transfected with p-sE1, p-sE2, p-sE1E2, p-sE1-IMX313P, p-sE2-IMX313P, or p-sE1E2-IMX313P and 50 μg of protein was analyzed for antigen expression in 10–12% (v/v) SDS-PAGE under reducing or non-reducing conditions as described previously (55–57, 59, 62). Mouse anti-E1 (MyBiosource, Cat. No. MBS310203) and human anti-E2 MAb HCV1 were used as primary antibodies to detect E1 and E2 expression followed by followed by anti-mouse-AF488 and anti-human AF555 (both from Invitrogen). The membrane was imaged using the LAS4000 digital imaging system (Fujifilm) and the resultant images were overlaid using ImageJ software (National Institutes of Health, USA) to obtain colocalization data.

### Protein Purification

After transfection of HEK293T cells with p-sE1E2-Histag, the histidine-tagged recombinant sE1E2 complex was purified by affinity chromatography from the cell culture medium using a Nickel-agarose column (Ni-NTA column; Qiagen) according to the manufacturer's recommendations. Briefly, the cell culture fluids were collected and clarified 48 h post-transfection, followed by ultrafiltration through a 70,000-molecular-weight cut-off filter (Amicon). The concentrated proteins were applied to the Ni-NTA column. Following three washes with buffer B (30 mM imidazole, pH 8), purified protein was eluted with 300 mM imidazole elution buffer (pH 8). Purified sE1E2 proteins were quantified using the Bradford protein assay (Bio-Rad) and analyzed by PAGE and Western blot.

### Animal Immunizations

Six to eight weeks old female BALB/c mice (*n* = 7 per group) were obtained from University of Adelaide Laboratory Animal Services and maintained under PC2 conditions in individually ventilated HEPA-filtered cages (Aero 80; Tecniplast, Italy) at the Queen Elizabeth Hospital animal facility. All experiments were conducted according to guidelines and protocols approved by the University of Adelaide Animal Ethics Committee (AEC; approval numbers: M-2018-30 and M-2015-135) and SA Pathology AEC (approval numbers: 2–18 and 29–15). For immunizations, mice were anesthetized with isoflurane and received 50 μg of total DNA per dose per animal of endotoxin-free DNA injected intradermally (ID) as described previously (51, 52). This is because the ID route is more effective than intramuscular or even subcutaneous DNA delivery [reviewed in (63)], likely due to a higher proportion of dendritic cells (DC) in the dermis/subdermis than other anatomical sites (63, 64). The DNA vaccines were administered 5–6 times at 3-weeks intervals.

### ELISpot Analysis

The frequency of mouse IFN-γ secreting E1 or E2-specific T cells was measured by ELISpot assay as described previously (49, 51). Briefly, red blood cell-depleted splenocytes from vaccinated mice, at 5 × 10^5^ cells per well, were stimulated with 4 μg/ml of either E1 or E2 peptides (HCV genotype 1b J4 Peptides, E1 catalog number: NR3738, E2 catalog number: NR3739, provided by the National Institutes for Health Biodefense and Emerging Infectious Research Resources Repository (NIAID), Bethesda, MD) for 36 h at 37°C. The E1 peptides consisted of one pool of 28 overlapping 15–19 mer peptides spanning the entire E1 protein. The E2 peptides were divided into 2 pools containing 28 (E2_1−186_) and 27 (E2_176−363_) overlapping 15–19 mer peptides, respectively. Secreted IFN-γ was detected using anti-mouse IFN-γ-biotin (clone R4-6A2; MabTech), streptavidin-AP, and SigmaFast BCIP/NBT (Sigma). An ELIspot reader (AID Germany) was used to count the spots. To generate the number of specific spot-forming units (SFU) per 10^6^ cells, the number of spots in unstimulated splenocytes was subtracted from the number of spots in the peptide-stimulated splenocytes. Data are presented as mean ± the standard error of the mean (SEM). Responses to E2 peptide pool 1 and 2 were combined to give a cumulative value for E2.

### ELISA for HCV E1/E2 Specific IgG

Nunc MaxiSorp ELISA plates were coated with 2 μg Ni-NTA Agarose chromatography purified sE1E2 proteins in 50 μl 0.1 M sodium bicarbonate buffer overnight at 4°C. The wells were then blocked with 2.5% BSA/PBS (blocking buffer) for 2 h at 37°C and washed four times with 0.01% Tween 20 in PBS (PBS-T). Mouse sera were serially diluted in blocking buffer then added to the plate overnight at 4°C, followed by four washes in PBS-T. The Secondary antibody, goat anti-mouse immunoglobulin (IgG) HRP (Invitrogen), was diluted 1: 3,000 in blocking buffer and added to the plate for 1 h at 37°C, followed by four washes with PBS-T. 1-Step™ Ultra TMB-ELISA substrate (ThermoFisher Scientific) was added and the reaction was stopped with 2 M sulfuric acid. The absorbance was measured at 450 nm using a FLUOstar OPTIMA plate reader (BMG LabTech). HCV E1 and E2-specific IgG endpoint titers were determined as the highest reciprocal dilution of serum with an optical density (OD) reading above the cut-off, set as 2 standard deviations (SD) above the mean OD of serum samples from pVax-vaccinated mice.

### Peptide ELISA for Mapping of E1 and E2 Epitopes

HCV peptide ELISA was performed using overlapping peptides representing the E1 or E2 region of the HCV J4 isolate (gt1b) as described previously (39, 65). The peptides consisted of 18-mers with an overlap of 11 amino acids covering the E1 or E2 HCV glycoprotein region (BEI Resources, NIAID, Bethesda, MD). Briefly, plates were coated with 5 μg/ml of 4–5 pooled peptides per well, blocked with 4% BSA/PBS, incubated with mouse sera (1:50 dilution) for 4 h at 37°C and antibody binding was assessed using an ELISA setup as described above.

### HCV Neutralization of Binding Assay

The HCV neutralization assay to measure the inhibition of HCV VLP binding and entry into Huh7 cells was performed as described previously (66, 67). Briefly, 50 ng of fluorescein isothiocyanate (FITC) labeled gt1b HCV-VLPs were incubated with serial dilutions of sera from vaccinated or non-vaccinated mice for 1 h at 37°C. The complex was then allowed to bind to Huh 7 cells for 1 h at 4°C, then incubated at 37°C for 1 h to promote entry. The cells were then washed to remove unbound HCV-VLPs and fixed in BD Cytofix (Becton Dickinson, USA). Inhibition of VLP binding/entry was determined by flow cytometry using FACS Calibur flow cytometer (Becton Dickinson) and analyzed using WinMDI II software. As a positive control, inhibition of entry of FITC-HCV VLPs into Huh7 cells was also determined using an anti-CD81 antibody (Abcam). Normal mouse serum was used as a negative control.

### HCVpp Production and Neutralization Assay

Retroviral HCV pseudoparticles (HCVpps) were prepared and the neutralization assay performed as described previously (68). Briefly, heterologous HCVpp harboring HCV envelope glycoproteins representing the 6 main HCV genotypes including H77.20 (GenBank accession AF011751), UKN1A20.8 (EU155192), UKN1B5.23 (AY734976), UKN2A1.2 (AY734977), UKN2A2.4 (AY734979), UKN2B1.1 (AY734982), UKN2B2.8 (AY734983), UKN3A1.28 (AY734984), UKN3A1.9 (AY734985), UKN3A13.6 (AY894683), UKN4.11.1 (AY734986), UKN4.21.16 (AY734987), UKN5.14.4 (AY785283), UKN6.5.8 (EF427671), and UKN6.5.340 (AY736194) were titrated to standardize the infectivity of all HCVpp to be 5 to 20-fold more infectious than mock pseudoparticles lacking HCV E1/E2. Mouse serum samples diluted at 1:5 or 1:10 were incubated with equivalent amounts of the titrated HCVpp for 1 h at 37°C and added to Huh7.5 cells (Apath, New York, NY, USA). The cells were lysed after 72 h and Bright Glo reagent (Promega) was added, followed by luminescence measurement on a CLARIOstar microplate reader (BMG Labtech). The percentage neutralization was calculated as (1 – RLU_test mouse serum_÷RLU_pre−immune mouse serum_) × 100.

### Statistical Analyses

Data are presented as means ± the standard errors of the mean (SEM). Statistical analysis was performed using unpaired Mann-Whitney tests, with ^*^P ≤ 0.05, ^**^P ≤ 0.01, ^***^P ≤ 0.001, and ^****^P ≤ 0.0001 considered significant. The analysis was performed using GraphPad Prism 7 for Windows (GraphPad Software, La Jolla, CA).

## Results

### Secreted HCV-E1/E2 Proteins Form Oligomers Following Fusion to IMX313P

DNA vaccines, each encoding a different form of sE1 and/or sE2, were constructed in pVAX: p-sE1, p-sE2, p-sE1E2, p-sE1-IMX313P, p-sE2-IMX313P, and p-sE1E2-IMX313P (Figure 1A). To confirm expression, secretion and oligomerization of E1 and E2, cell culture supernatant fluids were collected from transfected HEK293 cells and western blot analysis was performed under reducing (Figure 1B) and non-reducing (Figure 1C) conditions. The protein expressed from the p-sE1E2-Histag construct that was used to coat ELISA plates was purified using a Nickel-agarose column before western blot analysis (Figure 1B).

Oligomers of sE1- and/or sE2-IMX313P were only detectable in non-reducing conditions and accordingly, under reducing conditions, proteins that migrated as monomers of 37 kDa for E1 and ~68 kDa for E2 were detected using E1 and E2-specific antibodies (Figure 1B). As the coding sequences for the E1 and E2 TMDs were removed from the DNA constructs and the proteins no longer contained a canonical protease cleavage site, the sE1E2 proteins were not cleaved into E1 and E2 but migrated as a sE1E2 complex of ~80 kDa (Figure 1B). The sE1 and/or E2 proteins fused to IMX313P fusion proteins migrated under non-reducing conditions as a broad oligomeric band of molecular mass >250 kDa (Figure 1C). As expected, oligomers were not detected in supernatant fluids from cells transfected with p-sE1, p-sE2, p-sE1E2, or p-sE1E2-Histag (Figure 1C). These proteins migrated under non-reducing conditions as monomers. No HCV-specific bands were detected from proteins isolated from cells transfected with the pVax control. The expression of E1 and E2 was also assessed by immunofluorescence (Supplementary Figure S1).

Folding of these different forms of the HCV envelope proteins was assessed by a set of human MAbs including HC33.1 (69), HCV1 (70), AR3C (71), 2A12 (72), and HC84.27 (73) (kindly provided by Heidi Drummer), capable of binding to the E2 protein (Figure 1D). These MAbs, except 2A12, block the interaction of E2 with CD81 by binding to the regions of E2 that form the CD81 receptor binding site (35, 69–73) and demonstrated that the respective neutralizing epitopes were exposed following protein expression. As expected, these MAbs were unable to bind to cells transfected with constructs encoding E1 only (Figure 1D). These results demonstrate that fusion of IMX313P to sE1, sE2, or sE1E2 resulted in oligomerization of each of these proteins and confirmed that the different forms of the envelope proteins encoded in pVax were expressed and detected as predicted.

### DNA Vaccines Encoding E1E2 Fused to IMX313P Generate Significant T-Cell Immune Responses *in vivo*

Groups of mice were vaccinated with (i) p-sE1 + pVax, (ii) p-sE2 + pVax, (iii) p-sE1 + p-sE2, (iv) p-sE1-IMX313P + pVax, (v) p-sE2-IMX313P + pVax, (vi) p-sE1E2-IMX313P + pVax, or (vii) p-sE1-IMX313P + p-sE2-IMX313P. pVax only vaccinated mice served as a mock control and pVax was included in some vaccine cocktail regimens to ensure that all animals received an equimolar amount of DNA.

**Figure 2:**
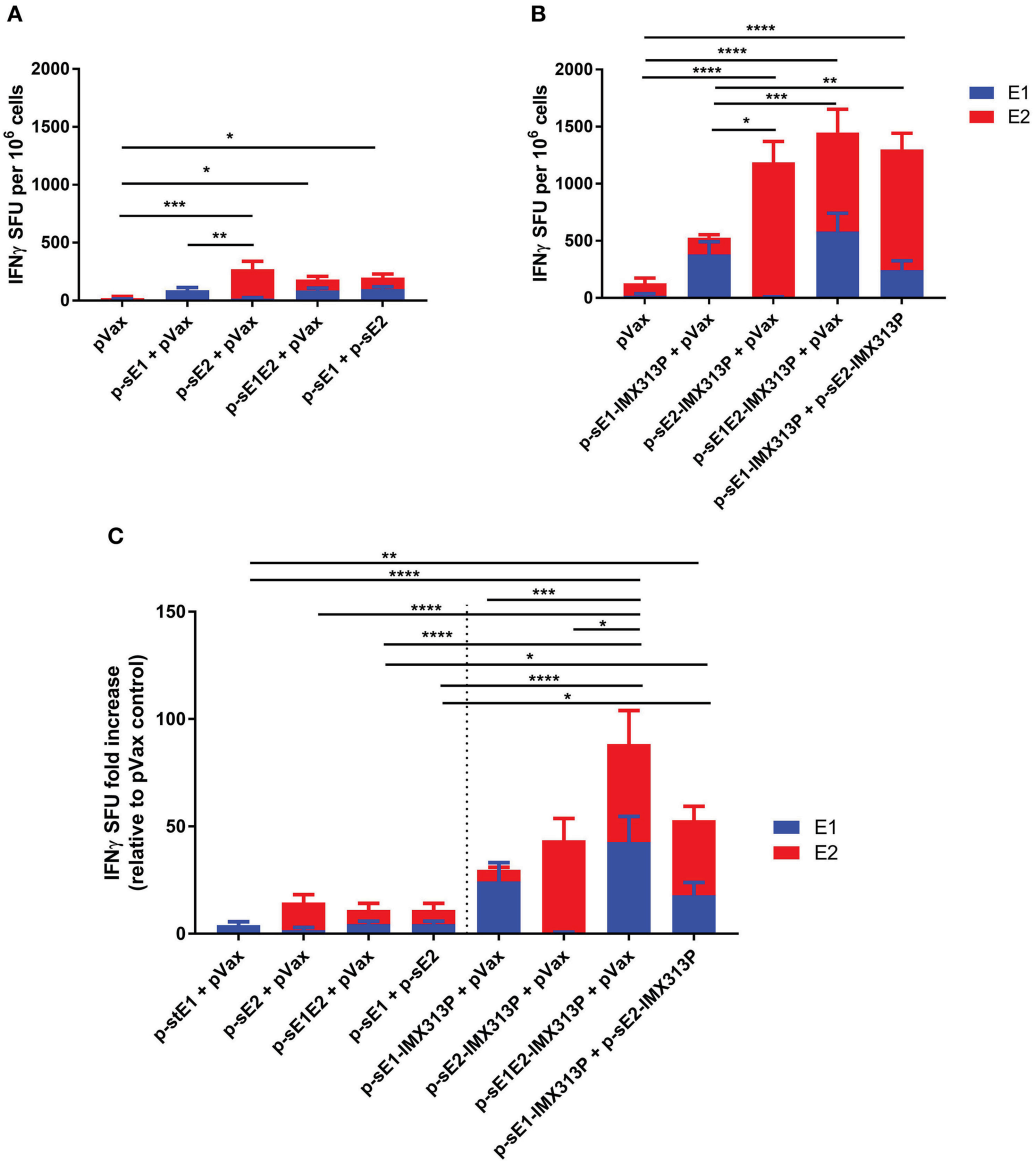
IMX313P adjuvanted DNA vaccination elicits higher HCV-specific cellular immune responses in BALB/C mice. Mice were vaccinated six times at 3-week intervals with DNA constructs encoding **(A)** sE1, sE2, sE1E2, **(B)** sE1 and/or sE2 fused to IMX313P. Splenocytes were harvested 28 days after the final vaccination, re-stimulated in duplicate with overlapping HCV peptide pools representing gt1b E1 and E2 proteins and IFN-γ secretion was measured by ELISpot assay. The data are expressed as mean (*n* = 7) SFU per 10^6^ cells responses to different peptide pools and presented as the mean ± SEM per group. The number of SFU in unstimulated cells was subtracted from the number in cells stimulated with peptides to generate the net HCV response. **(C)** ELISpot data plotted as mean SFU fold increase (+SEM) relative to pVax control for comparison. Significance tested against respective controls. *p ≤ 0.05; **p ≤ 0.01; ***p ≤ 0.001; and ****p ≤ 0.0001 (Mann-Whitney test non-parametric *t*-test).

Interferon (IFN)-γ enzyme-linked immunospot (ELISpot) assay was conducted to assess the induction of cellular immune responses against the E1 and E2 proteins. Four weeks after vaccination, splenocytes were harvested and stimulated with E1 and E2-specific peptides. Mice vaccinated with p-sE1, p-sE2, or p-sE1E2 resulted in the lowest IFN-γ spot forming unit (SFU) responses (Figure 2A), while mice vaccinated with the constructs encoding sE1/sE2 fused to IMX313P induced the highest IFNγ SFU responses (Figure 2B). Although the p-sE1-IMX313P + pVax group elicited minor responses to E2 peptides, this was not significant and can be assumed to represent background. As the data in Figures 2A,B were obtained in independent experiments, we pooled the data from these two experiments and calculated the IFN-γ SFU in each animal vaccinated with pVax encoding E1 and/or E2 as the fold increase over the mean responses observed in the respective pVax control group (Figure 2C). This analysis revealed that vaccination with p-sE1E2-IMX313P (+ pVax) elicited the greatest CMI responses to E1 and E2. In general, fusion of IMX313P to sE1, sE2, or sE1E2 in DNA vaccines enhanced the frequency of IFN-γ secreting T cells compared to the respective DNA vaccine counterparts lacking IMX313P (Figure 2C). However, statistically significant differences were only obtained when comparisons were made between p-sE1E2 + pVax vs. p-sE1E2-IMX313P + pVax and p-sE1 + psE2 vs. p-sE1-IMX313P + p-sE2-IMX313P (Figure 2C). Overall, the results shown in Figure 2 revealed that vaccination with a DNA construct encoding sE1 and/or sE2 proteins fused to the IMX313P domain induced superior cell mediated immune responses with the most immunogenic vaccine being p-sE1E2-IMX313P.

### Vaccination With DNA Encoding sE1 and/or sE2 With IMX313P Induces Superior Antibody Responses in Mice

**Figure 3:**
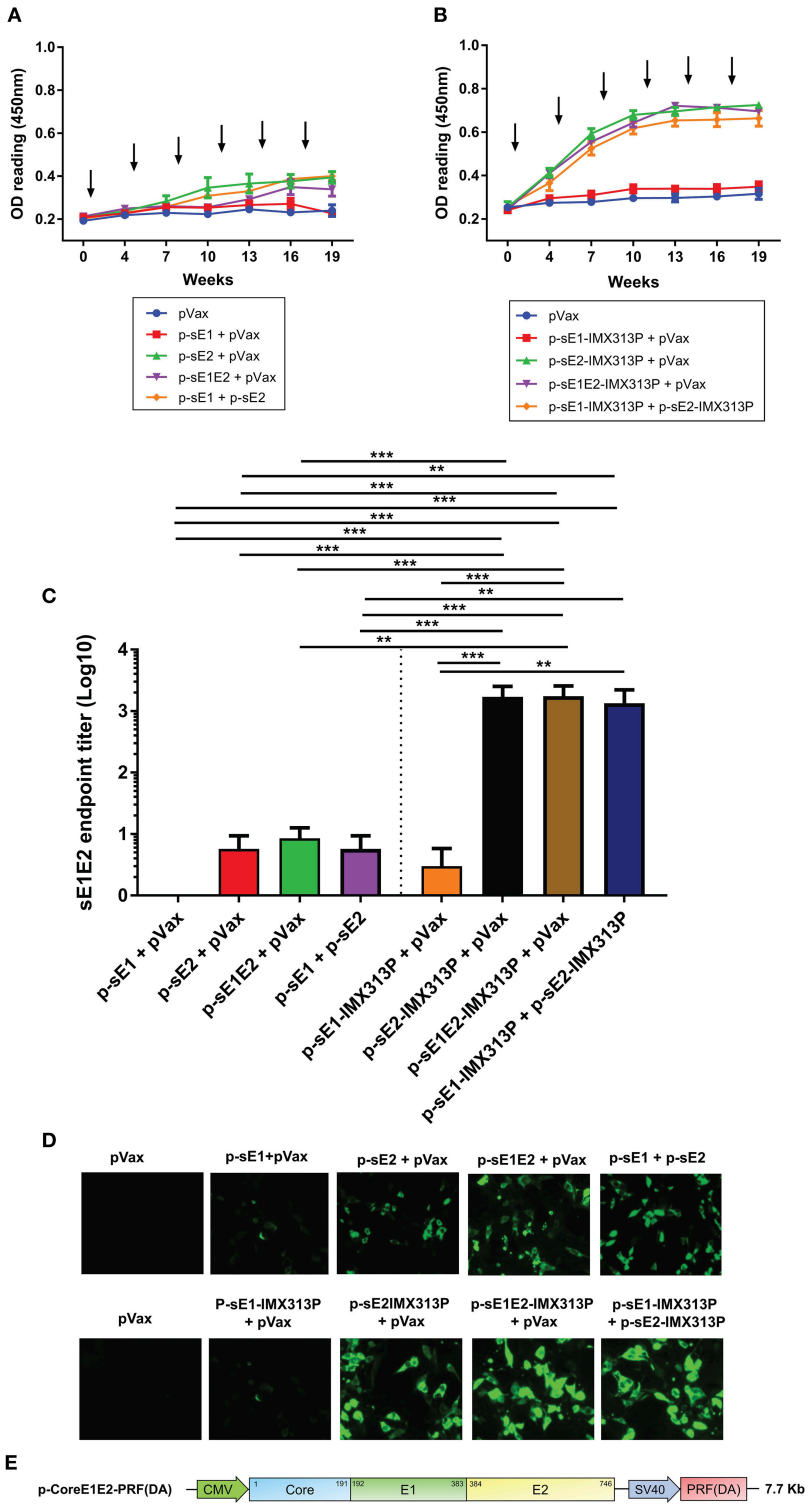
IMX313P adjuvanted DNA vaccination also induces higher HCV-specific antibody responses. Serum from mice vaccinated with **(A)** p-sE1, p-sE2, or p-sE1E2 or **(B)** p-sE1-IMX313P, p-sE2-IMX313P, or p-sE1E2-IMX313P was diluted (1/50) and anti-E1E2-specific antibodies were measured by ELISA at the indicated time points. The arrows indicate when the vaccines were administered. **(C)** sE1E2-specific serum IgG titers 4 weeks after the final immunization (week 19). Titers are expressed as the reciprocal of the serum dilution and plotted as Log10. The data represent mean responses in each group (*n* = 7) ± SEM. **p < 0.01, and ***p < 0.001 (Mann-Whitney non-parametric *t*-test). **(D)** Immunofluorescent analysis of antibodies specific for full-length E1 and E2 proteins in immune sera. HEK293T cells were transfected with a bicistronic construct, pVax-coreE1E2-PRF(DA) encoding full length gt1b core, E1 and E2 proteins and PRF(DA) (a non-cytolytic version of perforin containing a D483A mutation) (50–61); and pooled sera from immunized-mice were used as the primary antibody at a 1:50 dilution. **(E)** A schematic diagram of the pVax-coreE1E2-PRF(DA) DNA construct.

To determine the antibody responses elicited following vaccination with the different DNA vaccines, mouse serum was collected at intervals after each vaccination and analyzed for E1E2-specific antibodies by ELISA using affinity purified E1E2. As we showed that this E1E2 target contained both E1 and E2 proteins (Figures 1B,C), antibody responses detected by ELISA are referred to as anti-E1E2 responses. As shown in Figures 3A,B, the antibody levels peaked and plateaued following the 4th immunization for all groups although the antibody levels in the groups exposed to IMX313P were only modestly above those elicited by vaccines lacking IMX313P. However, vaccination with DNA constructs encoding E1 induced minimal levels of E1E2-specific responses. The anti-E1E2 antibody levels were higher in groups vaccinated with DNA encoding secreted envelope proteins and IMX313P compared to pVax, with the exception of mice vaccinated with p-sE1-IMX313P + pVax (Figure 3C). The end point titers for anti-E1E2 antibodies determined after the 4th immunization also support this trend and showed that vaccination with p-sE1-IMX313P + p-sE2-IMX313P resulted in the highest average titer of 1/1749, followed by p-sE2-IMX313P-vaccinated mice (average titer 1/1707), and p-sE1E2-IMX313P (average titer 1/1346). By comparison, vaccination with p-sE1E2, p-sE2 or p-sE1 induced titers of 1/113, 1/67, and 1/18, respectively (Figure 3C). Vaccination with p-sE1 or p-sE1-IMX313P induced the lowest antibody titers (average titer <1/10; Figure 3C). Additionally, antibodies generated following vaccination with these DNA constructs were also capable of binding to the full-length E1E2 heterodimer as shown by immunofluorescence analysis (Figure 3D). Collectively, the data indicate that DNA vaccines encoding secreted envelope proteins and IMX313P can elicit robust HCV E1E2-specific antibody responses and that these responses were superior to those induced by immunization with DNA vaccines encoding secreted envelope proteins only.

### E1E2 Protein Boost Increases Anti-E1E2 Responses in Mice Vaccinated With DNA Vaccines Encoding Secreted Envelope Proteins and IMX313P

**Figure 4:**
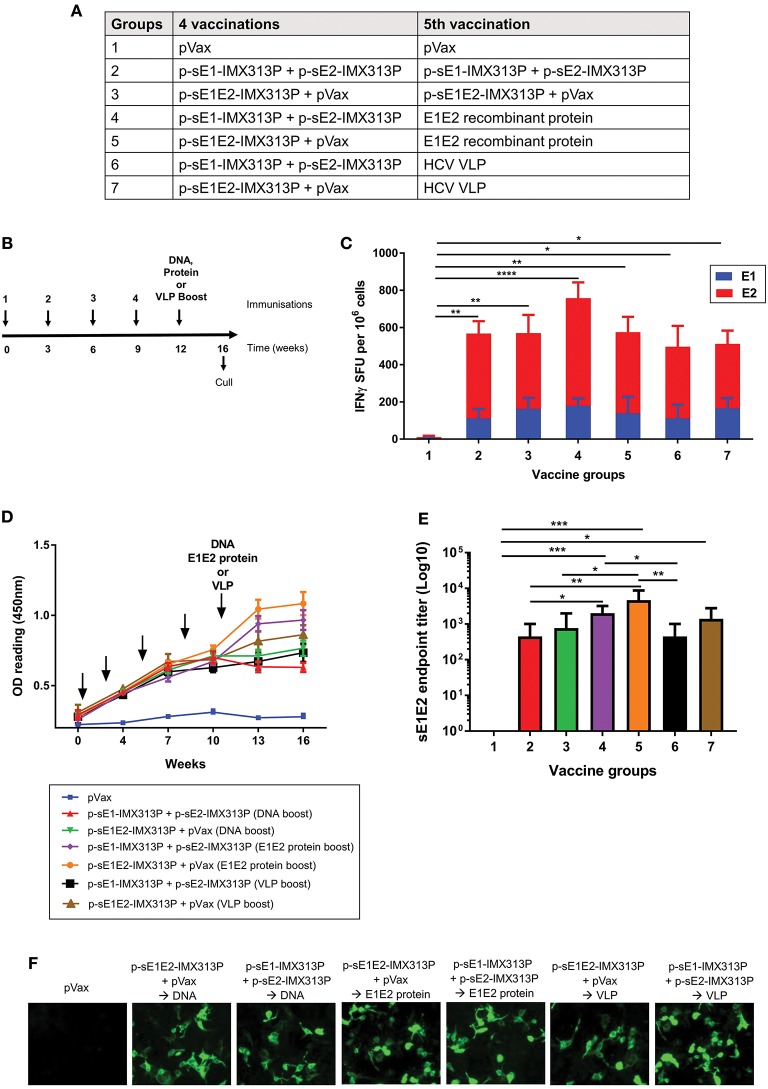
Boosting with recombinant sE1E2 protein augments the cellular and humoral immunity elicited in mice previously vaccinated with IMX313P adjuvanted DNA vaccines encoding secreted envelope proteins. **(A)** Vaccination groups and **(B)** immunization schedule of animals. The arrows indicate when the vaccines were administered. Female BALB/C mice (*n* = 7/group) were vaccinated at 3 weeks intervals via the ID route with 50 μg of p-sE1E2-IMX313P or p-sE1-IMX313P + p-sE2-IMX313P and boosted with DNA or E1E2 recombinant protein or HCV VLPs 3 weeks later. pVax was included in the vaccine cocktail to ensure that all animals were vaccinated with an equimolar amount of DNA. **(C)** IFN-γ ELISpot assay to examine E1- or E2-specific CMI in splenocytes stimulated with E1- or E2-peptides for 36 h. **(D)** Anti-E1E2-specific antibodies levels measured by ELISA (at 1:50 dilution) at the indicated time points. **(E)** sE1E2-specific serum IgG 4 weeks after the final immunization. Titers are expressed as the reciprocal of the dilution factor of the serum and plotted as Log_10_. The data represent mean responses in each group (*n* = 7) ± SEM. *p < 0.05, **p ≤ 0.01, ***p ≤ 0.001; and ****p ≤ 0.0001 (Mann-Whitney non-parametric *t*-test). **(F)** Immunofluorescence analysis of antibodies specific for full-length E1 and E2 proteins in immune sera. HEK293T were transfected with pVax-coreE1E2 encoding full length E1E2 proteins and pooled sera from mice immunized were used as the primary antibody at a 1:50 dilution.

The above studies suggest that the p-sE1-IMX313P + p-sE2-IMX313P and p-sE1E2-IMX313P vaccination regimens were the most robust in eliciting CMI and antibodies against E1E2. Next, we evaluated whether the immunogenicity of these vaccination regimens could be improved following booster immunizations with sE1E2 protein or VLPs expressing E1E2 (Figures 4A,B). The purified sE1E2 poly-His-tagged fusion protein described above constituted the E1E2 protein boost. The production and purification of the genotype 1 (gt1) HCV VLPs have been described elsewhere (66, 67). As shown above in Figures 3A,B, the antibody response plateau was reached following four DNA vaccinations, therefore mice were vaccinated with four doses of p-sE1-IMX313P + p-sE2-IMX313P or p-sE1E2-IMX313P, before receiving a boost with DNA, sE1E2 protein or the VLPs (Figure 4B).

To compare CMI elicited by the different regimens, the IFN-γ ELISpot assay was performed using E1 and E2 peptide pools to stimulate the splenocytes (Figure 4C). As shown in Figure 4C, comparable IFN-γ responses significantly above the pVax control were observed after all vaccination regimens (Groups 2–7) which encoded secreted envelope immunogens. However, p-sE1-IMX313P + p-sE2-IMX313P prime/E1E2 protein induced the highest responses, 178.5 SFU/10^6^ cells, following stimulation with E1 peptides and 579.3 SFU/10^6^ cells following stimulation with E2 peptides (Figure 4C), although these were not statically significant compared to the other vaccine groups.

To determine the antibody responses, mouse serum samples were collected and analyzed for E1E2-specific antibodies by ELISA. As previously observed, the antibody levels peaked and plateaued following the 4th immunization in all groups except for the pVax group (Group 1) which as expected did not elicit specific antibodies to E1E2 (Figure 4D). Vaccination with p-sE1E2-IMX313P followed by a boost with E1E2 protein (Group 5) resulted in the highest E1E2-specific antibody levels, although these levels were similar to those observed for the p-sE1-IMX313P + p-sE2-IMX313P prime/ E1E2 protein boost (Group 4) followed by the p-sE2-IMX313P prime/ HCV VLP boost (Group 7) (Figure 4D).

The antibody titers were measured 4 weeks post the final vaccination and showed that the DNA prime/E1E2 protein boost vaccination regimen induced significantly higher titers compared to boosting with DNA or the HCV VLPs (Figure 4E). Boosting with the sE1E2 proteins following immunization with p-sE1E2-IMX313P or p-sE1-IMX313P + p-sE2-IMX313P resulted in the highest anti-E1E2 antibody responses (average titer of 1/4,678 and 1/2,004, respectively) (Figure 4E). This was followed by responses from animals vaccinated with p-sE1E2-IMX313P and boosted with VLPs (average titer: 1/1,386) or p-sE1E2-IMX313P DNA (average titer: 1/768). Animals vaccinated with p-sE1-IMX313P + p-sE2-IMX313P and boosted with the same DNA or HCV VLPs generated similar anti-E1E2 responses with an average titer of 1/446 (Figure 4E). These antibodies were also capable of binding to full length E1E2 as shown by immunofluorescence (Figure 4F). Collectively, the data indicate that recombinant E1E2 protein provides a superior boost compared to DNA or HCV VLPs and is capable of enhancing HCV E1E2-specific antibody responses in p-sE1E2-IMX313P or p-sE1-IMX313P + p-sE2-IMX313P-vaccinated mice.

### Prime-Boost Vaccinations With Vaccines Encoding or Expressing Monomeric or Oligomerized Secreted Envelope Proteins Elicit Antibodies That Recognize E1 and E2 Epitopes

**Figure 5:**
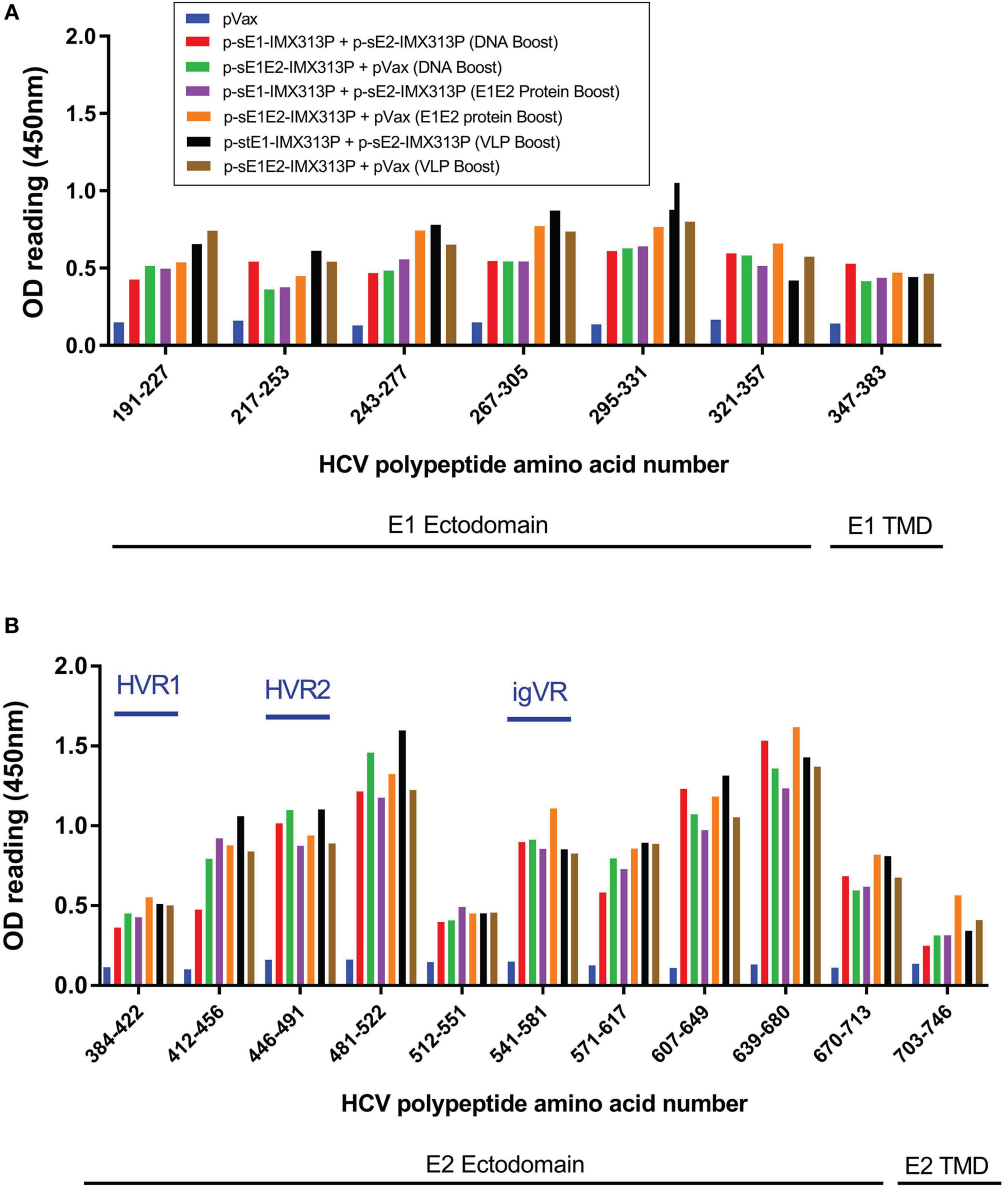
Reactivity of sera from DNA prime/DNA boost, DNA prime/E1E2 protein boost or DNA prime/HCV VLP boost-immunized animals to E1E2 peptides. Serum antibody responses specific to a library of overlapping **(A)** E1 or **(B)** E2 peptides, supplied by BEI Resources, in ELISA 4 weeks after the last vaccination. Serum from vaccinated mice was diluted (1/50) and added to wells coated with HCV peptides. Values represent mean responses in each group (*n* = 7) + SEM. Data are representative of 3 independent experiments performed in duplicate.

Overlapping peptide ELISA was conducted to map the reactivity of serum antibodies to continuous and discontinuous epitopes on E1E2. ELISA plates were coated with E1 or E2 overlapping peptides to assess the serum antibody responses to continuous epitopes (Figure 5). There was little difference in reactivity to the E1 peptides between all the immunized groups, although sera from animals immunized with the p-sE1E2-IMX313P DNA prime/ E1E2 protein boost regimen or the DNA prime/ HCV VLP boost regimen showed a slightly higher reactivity compared to other immunized groups (Figure 5A). In contrast, the sera showed greater reactivity against E2 and reacted with peptides spanning aa 412–522 and aa 541–713 (Figure 5B), while the highest reactivity was seen toward aa 481–522, 607–649, and 639–680. All vaccinated groups showed limited reactivity toward hypervariable region (HVR) 1, aa 384–411, and the TMD, aa 716–747, but some reactivity against HVR2, aa 460–485, and aa 570–580 representing the intergenotypic variable region (igVR; also known as HVR3) (Figure 5B). As the TMD was not included in the vaccine immunogen, the level of reactivity against peptides representing this region can be assumed to represent background.

### A DNA Boost Induced Cross-Neutralizing Antibodies in Mice Vaccinated With p-sE1/sE2-IMX313P

**Figure 6:**
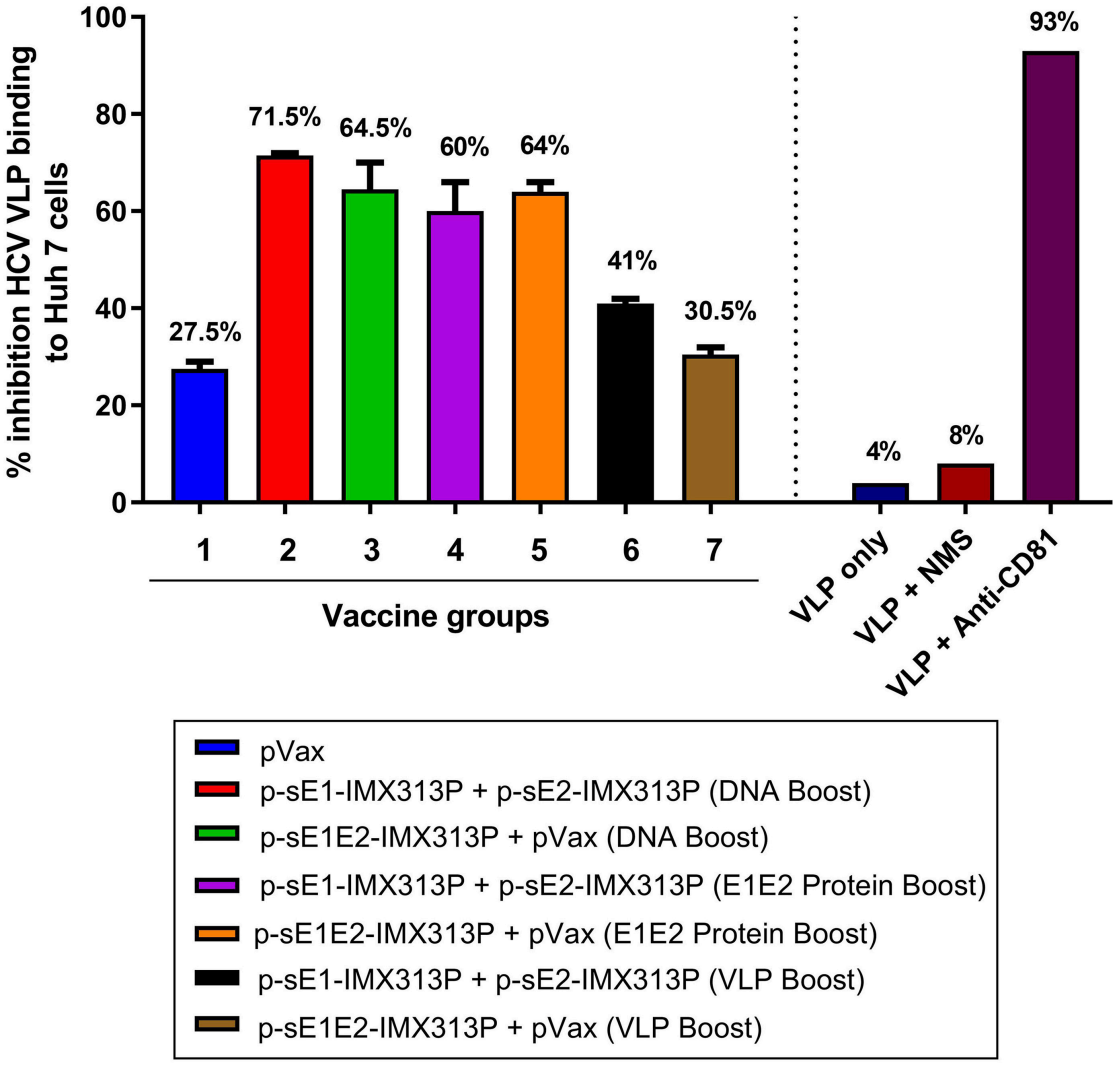
p-sE1/sE2-IMX313P DNA prime/DNA boost vaccination elicits anti-E1E2 antibodies with HCV VLP neutralizing activity. Neutralization of VLP binding was determined by pre-incubating fluorescent-labeled VLPs with pooled serum from vaccinated mice followed by incubation with Huh7 cells. The cells were then harvested, and fluorescence levels analyzed by flow cytometry. All bar graphs show the percentage reduction in VLP entry following incubation with mouse sera at 1:25 dilution. The percentage values are indicated at the top of each bar. Y-axis depicts the percentage inhibition of binding of HCV-LPs to Huh7 cells; X-axis represents the different vaccine or control groups.

To assess the neutralizing ability of the antibodies generated by the different vaccination regimens, we investigated the ability of the sera to inhibit binding of genotype 1b HCV VLPs to Huh7 cells. Fluorescent-labeled HCV VLPs were incubated with the serum at 1/5 and 1/25 dilutions, and the binding of the VLPs to Huh7 cells was monitored by flow cytometry as described (66, 67). Incubation of the HCV VLPs with PBS (VLP only) or normal mouse serum (NMS; Sigma) resulted in minimal inhibition of HCV VLP binding to hepatocytes (8%), whereas the addition of anti-CD81 antibodies on the other hand (Figure 6), inhibited VLP binding to Huh7 cells by >90% confirming that these particles use CD81 to bind to target cells, in a similar manner to HCV (74, 75).

Dose-dependent inhibition of binding of the HCV VLPs by serum from the vaccinated mice was observed across all vaccination groups although most of the samples showed some non-specific inhibition at a 1/5 dilution (data not shown). At a 1/25 dilution which was considered to represent specific inhibition, the highest neutralization of HCV VLPs binding to Huh7 cells was detected in group 2 with 71.5% neutralization (Figure 6). However, the neutralization levels were comparable between groups 2–5. Surprisingly the lowest inhibitions were observed in groups 6 and 7 with 41% and 30.5% neutralization, respectively (Figure 6). Although these results were not statistically significant, they suggest that antibodies generated against HCV genotype 1b E1/E2 proteins possess neutralizing properties and could neutralize the binding of genotype 1b VLPs to target cells, and that homologous DNA prime-boost vaccination with p-sE1-IMX313P + p-sE2-IMX313P (group 2) resulted in the highest neutralization activity to prevent HCV VLP binding/entry into hepatocytes.

Additionally, the mouse sera were tested for their capacity to neutralize HCVpp pseudotyped with envelope proteins from different genotypes including genotypes 1a, 1b, 2a, 2b, 3a, 4, 5, or 6 (Table 1). Although a range of serum dilutions was tested, only the 1/5 and 1/10 dilutions showed evidence of neutralization. Essentially, all anti-E1- and/or anti-E2- antibody positive samples showed dose-dependent cross-neutralizing properties *in vitro* against the different HCVpp genotypes and showed a minimum of 70% neutralization (at 1/5 dilution) against HCVpp from genotypes 1a, 1b, 2a, 3a, and 4 (Table 1). However, serum from these animals (groups 2–7) showed lower neutralizing activity against genotypes 2a, 2b, 4, and 6, and were less efficient at neutralizing HCVpp from genotypes 5 and 6 (Table 1). Serum from mice vaccinated with pVax generally showed minimum neutralization of the HCVpp except for pseudotyped particles from a few genotypes with the 1/5 serum dilution.

**Table 1:** Serum neutralization of HCVpp following immunization.

	**Group 1**		**Group 2**		**Group 3**		**Group 4**		**Group 5**		**Group 6**		**Group 7**	
	**1/5**	**1/10**	**1/5**	**1/10**	**1/5**	**1/10**	**1/5**	**1/10**	**1/5**	**1/10**	**1/5**	**1/10**	**1/5**	**1/10**
HCVpp gt1a (H77.20)	8	15	69 ^(1)^	42 ^(1)^	80 ^(1, 3, 4, 6)^	54 ^(1)^	61 ^(1)^	38 ^(1)^	59	27	72 ^(1)^	39 ^(1)^	56	10
HCVpp gt1a (UKN1A20.8)	0	0	92 ^(1, 2)^	59 ^(1, 6)^	100 ^(all)^	70 ^(1, 6)^	91 ^(1)^	61 ^(1, 6)^	89 ^(1)^	52 ^(1, 6)^	94 ^(1)^	60 ^(1, 6)^	93 ^(1, 4)^	27
HCVpp gt1b (UKN1B12.16)	6	9	76 ^(1)^	51 ^(1)^	89 ^(all)^	62 ^(1, 4, 6)^	75 ^(1, 4)^	53	70 ^(1)^	47	80 ^(1, 3, 4)^	47	71 ^(1)^	48
HCVpp gt1b (UKN1B5.23)	0	0	91 ^(1)^	66 ^(1, 3, 4, 5)^	96 ^(1, 4, 5, 6)^	64 ^(1, 4, 6)^	93 ^(1, 4, 5, 6)^	55 ^(1)^	83 ^(1)^	53	90 ^(1, 3, 4)^	53 ^(1)^	90 ^(1, 4)^	43
HCVpp gt2a (UKN2A1.2)	25	24	77 ^(1)^	42 ^(1, 4, 5, 6)^	84 ^(1, 3, 4, 6)^	49 ^(1, 4, 5, 6)^	80 ^(1, 4)^	42 ^(6)^	72 ^(1)^	30	78 ^(1)^	26	76 ^(6)^	15
HCVpp gt2a (UKN2A2.2)	0	0	53 ^(1, 4)^	31 ^(1, 6)^	60 ^(1, 4, 6)^	24 ^(1)^	39 ^(1)^	6	26 ^(1)^	0	58 ^(1, 4)^	10	33 ^(1)^	0
HCVpp gt2b (UKN2B1.1)	0	0	72 ^(1, 4, 5, 6)^	38 ^(1, 4)^	69 ^(1, 4, 6)^	38 ^(1, 4, 5)^	52 ^(1)^	39 ^(4, 5)^	35 ^(1)^	18	46 ^(1, 6)^	30 ^(4)^	30 ^(1)^	27
HCVpp gt2b (UKN2B2.8)	0	0	78 ^(1, 3)^	34 ^(1)^	78 ^(1, 3)^	34 ^(1)^	63 ^(1)^	25 ^(1)^	65 ^(1)^	25 ^(1)^	67 ^(1)^	21 ^(1)^	72 ^(1, 3)^	23 ^(1)^
HCVpp gt3a (UKN3A1.28)	62	0	94 ^(1)^	33	92 ^(1)^	27	97 ^(1, 6)^	28	85 ^(1)^	19	95 ^(1, 6)^	0	89 ^(6)^	29
HCVpp gt3a (UKN3A1.9)	36	5	92 ^(1, 4, 6)^	48 ^(1, 5, 6)^	98 ^(all)^	63 ^(1, 4, 5, 6)^	90 ^(1, 6)^	36	85 ^(1)^	40 ^(6)^	88 ^(1, 6)^	28	86 ^(1)^	26
HCVpp gt3a (UKN3A13.6)	0	0	87 ^(1, 4)^	63 ^(1)^	95 ^(all)^	70 ^(1, 3, 5, 6)^	86 ^(1, 4)^	41 ^(1)^	78 ^(1)^	47 ^(1)^	87 ^(1, 4)^	43 ^(1)^	87 ^(1, 4)^	37 ^(1)^
HCVpp gt4 (UKN4.11.1)	0	0	94 ^(1)^	55 ^(1, 4)^	97 ^(1, 3, 4, 5)^	62 ^(1, 4)^	88 ^(1)^	55 ^(1, 4)^	79 ^(1)^	40 ^(1)^	84 ^(1)^	41 ^(1)^	97 ^(1, 3, 4, 5)^	51 ^(1, 4)^
HCVpp gt4 (UKN4.21.16)	32	36	63 ^(1)^	52 ^(1, 4, 6)^	78 ^(1, 4, 5, 6)^	51 ^(1, 3, 4, 5, 6)^	62 ^(1)^	41	60 ^(1)^	42 ^(6)^	63 ^(1)^	55 ^(1, 3, 4, 6)^	57 ^(1)^	33
HCVpp gt5 (UKN5.14.4)	42 ^(4)^	10	41	5	57 ^(4, 6)^	0	31	0	2	0	11	0	0	0
HCVpp gt6 (UKN6.5.8)	8	20	75 ^(1, 4, 6)^	48 ^(1, 4, 6)^	78 ^(1, 4, 6)^	46 ^(1)^	70 ^(1)^	40 ^(1)^	64 ^(1)^	34 ^(1)^	72 ^(1, 6)^	40 ^(1)^	52 ^(1)^	30
HCVpp gt6 (UKN6.5.340)	22	26	47 ^(1, 6)^	35	51 ^(1, 6)^	35	37	21	17	11	33	17	0	6

*Serum samples collected 4 weeks post the last vaccination were tested at dilutions of 1/5 (Gray background) and 1/10 (white background) for their HCVpp neutralizing properties. The assay was performed in quadruplet, and the data shown are the mean percent neutralization. Statistical analyses were done using Mann-Whitney (non-parametric) t-test by comparing the neutralization observed between the vaccine groups at the respective dilutions. Significance are shown in parentheses: (1) denotes significance from group 1 (p ≤ 0.05), (2) denotes significance from group 2 (p ≤ 0.05), (3) denotes significance from group 3 (p ≤ 0.05), (4) denotes significance from group 4 (p ≤ 0.05), (5) denotes significance from group 5 (p ≤ 0.05), (6) denotes significance from group 6 (p ≤ 0.05), (all) denotes significance from all groups (p ≤ 0.05)*.

Similar to the results observed in Figure 6, vaccination with the homologous DNA prime/DNA boost regimen induced the highest NAb responses (Groups 2 and 3, Table 1). Antibodies generated after vaccination with p-sE1E2-IMX313P were the most effective at cross-neutralizing the HCVpp with >50% neutralization at 1/5 serum dilution across all genotypes (Group 3, Table 1). The cross-neutralizing properties of antibodies generated following vaccination with the DNA prime/ VLPs boost were generally similar to those produced by vaccination with the DNA prime/ E1E2 protein boost, although the latter elicited higher antibody titers. However, at 1/10 dilution, the sera from DNA prime/VLPs boost vaccinated mice were generally less efficient at neutralizing the HCVpp compared to sera from mice that received the DNA prime/E1E2 protein boost regimen. In contrast, the antibodies generated by the DNA prime/DNA boost regimen still showed high HCVpp cross-neutralizing activity at 1/10 dilution.

Overall, the data show that vaccination with a DNA vaccine encoding a secreted, oligomerized form of E1E2 can significantly augment E1E2-specific immune responses, and importantly generate antibodies with cross-neutralizing activity.

## Discussion

An effective vaccine is the optimal long-term, cost-effective solution to combat HCV globally. Studies in humans and non-human primates have provided insight into the possible correlates of protection, although the actual correlates remain poorly understood. It is generally accepted that an effective HCV vaccine should at least induce NAb targeting envelope proteins, but it is likely that CMI against the non-structural proteins will also be required. HCV envelope proteins are involved in essential initial steps in the virus replication cycle including viral attachment/entry or membrane fusion. Therefore, E1 and E2 represent significant vaccine immunogens and have been a major focus of various HCV vaccine development studies (17, 18, 66, 76, 77). Consequently, these proteins were the focus of the vaccine strategies described in this study. Previous reports have demonstrated that E1 and E2 glycoproteins expressed on the cell surface are more immunogenic than the wild-type antigens (41, 78) and secreted E1E2 proteins have also been shown to be significantly more immunogenic in mice (39). Antigen oligomerization has also been shown to improve vaccine immunogenicity (55–57). The work presented in this study aimed to develop a DNA vaccine encoding truncated E1 and E2 fused with the oligomerization domain of a chimeric C4 binding protein, IMX313P, that results in the production of secreted, oligomerized forms of the proteins. The immunogenicity of DNA vaccines encoding E1 and/or E2 glycoproteins as separate immunogens or as a single E1E2 polyprotein was assessed in mice.

While oligomerization may be an important element, the mechanism by which the C4b-p enhances immunogenicity is still not well-understood (55, 62). Therefore, in addition to oligomerization, other factors such as increased half-life, improved antigen uptake, and/or prolonged antigen processing could be involved in the enhancement of the E1/E2-specfic responses.

All groups immunized with the HCV DNA vaccines encoding E1E2 heptamers elicited significant levels of anti-E1E2 antibodies. This may be the result of increased capture and processing of the heptamers by both local and distal APCs, consequently increasing the immunogenicity of the E1E2 antigens (41, 55, 57, 79). Moreover, these antibodies were also able to recognize E1E2 proteins in their native (presumably heterodimeric) form as determined by immunofluorescence analysis of cells transfected with a DNA construct encoding the full-length coreE1E2 polyprotein (80, 81). However, only limited anti-E1-specific antibody responses were generated, in accordance with previous findings (18, 39, 43) suggesting that the observed antibody responses comprised mainly of anti-E2 antibodies. Nevertheless, the antibody titers generated by vaccination with E2-encoding constructs were comparable to those reported in the literature (66, 77, 82).

In addition to strong humoral responses, robust CMI responses have been reported to be critical for clearance of HCV in humans and chimpanzees during acute infection (83–86). Fusion of the envelope proteins to IMX313P resulted in superior E1 and/or E2-specific antibody and CMI responses compared to their monomeric counterparts. A robust CD8^+^ T cell response with IFN-γ^+^ production has also been associated with spontaneous clearance of HCV, and the impairment of this response is a characteristic feature of persistent infection (30, 85, 87–89). All groups immunized with the DNA vaccines encoding E1E2 heptamers induced significant IFN-γ responses compared to animals immunized with the monomeric DNA constructs as determined by ELISpot. The number of SFUs detected in the E1/E2-specific ELISpot reported in this study compared favorably with those reported in other studies using viral vectors (76, 77, 90), DNA (91), or VLPs (67, 91).

A DNA prime-protein boost strategy is a promising method to increase the effectiveness of DNA immunization (92). Vaccination with the DNA prime/DNA, E1E2 protein, or VLP boost vaccination regimens resulted in the induction of high levels of E1E2-specific antibody- and CMI- responses. Among the three immunization strategies, boosting with recombinant E1E2 protein induced the highest serum IgG levels. Thus, the sE1/sE2-IMX313P DNA prime/E1E2 recombinant protein boost vaccine strategy is consistent with current immunization regimens involving priming with DNA followed by boosting with viral vectors or a recombinant protein that have been favored to induce protective immune responses to many diseases, such as HCV, HIV and malaria (93, 94). Serum antibody characterization revealed that the hyperimmune sera showed greater reactivity toward epitopes located in the ectodomain of E2. The E2 HVR1 (aa 384–410) is an immunodominant region that has been reported to be involved in viral escape in infected chimpanzees and humans (95, 96). However, the mouse sera showed little reactivity against this region, similar to observation from previous reports (39, 41, 42), perhaps reflecting the nature of the heptamer.

To date, a majority of all effective viral vaccines work through the generation of NAb, typically by targeting viral envelope proteins (97). For HCV, complete neutralization of incoming virions is difficult to achieve as the virus can be transmitted as free virions or associated with different lipoproteins and immunoglobulins (98, 99). Additionally, the presence of a quasispecies with variable epitopes in the virus population is likely to contribute to evasion of the humoral response. Nevertheless, neutralization of a proportion of incoming HCV virions might lead to a reduced viral load allowing the timely development of CMI, promoting viral clearance. The neutralization assay (Table 1) data suggested that vaccination with DNA prime/DNA boost, notably p-sE1E2-IMX313P, induced the highest cross-NAb responses while vaccination with DNA prime/E1E2 protein boost or DNA prime/VLP boost regimens generally induced lower inhibition of binding of HCV VLPs to Huh7 cells as well as HCVpp neutralization. These results contradict previous findings which demonstrated that immunization with HCV VLPs induced HCV-specific NAb, since the envelope proteins are presented on the surface of VLPs in their correct conformation during immunization (66, 67). Our results also suggest that, despite achieving a plateau of antibody responses following four DNA vaccinations, an additional DNA vaccination was necessary to induce significant NAb, most likely reflecting the poor immunogenicity of the HCV envelope proteins. Furthermore, the single dose of VLPs administered in this study might have been inadequate as previous studies have demonstrated that two or more doses of VLPs are necessary to induce NAb (66). Therefore, it is possible that additional boosting with the VLPs or E1E2 proteins may have induced even higher NAb. Nevertheless, the neutralization levels reported in this study compared well with previous studies (39, 44, 66, 67, 91) at similar serum concentrations.

The novel p-sE1/sE2-IMX313P DNA vaccine constructs described in this study represent an attractive candidate for a HCV vaccine that could be readily scaled up. A recombinant HCV E1E2 glycoprotein (genotype 1a) vaccine was previously reported to decrease the carrier rate in vaccinated chimpanzees and induce protective immune responses in some persons following homologous and heterologous gt1a virus challenge (16, 100). Furthermore, other E1E2 candidate vaccines demonstrated broad NAb responses in immunized human volunteers (22, 101, 102). Secreted forms of E2 can be generated using various cell lines including mammalian and insect cells and is the focus of the development of a humoral HCV vaccine (103, 104). However, cross-neutralizing epitopes representing E1 and E2 residues are not expressed in secreted E2 proteins (105). Additionally, recombinant E1 protein (gt1b), not E2, protected vaccinated chimpanzees from homologous gt1b viral challenge (106). Finally, developing an E1E2-specific NAb response may be a perquisite to mount a successful CMI response for viral clearance (8, 107). For these reasons, a DNA vaccine encoding secreted E1-E2 and IMX313P remains a prime candidate for the development an HCV vaccine.

Thus, far, the correlates of protective immunity against HCV have not been fully defined. Nevertheless, it is generally accepted that an effective vaccine against HCV should induce a robust antibody response capable of neutralizing the virus as well as a potent, broad CMI, to restrict virus propagation and spread (108, 109). We have previously developed novel DNA vaccines capable of generating robust CD4^+^ and CD8^+^ T cell responses against NS proteins 3, 4A, 4B, and 5B (51, 52) similar to those observed during resolution of acute HCV infection. We also recently showed that a multigenotypic DNA cocktail vaccine encoding gt1b NS5B proteins and gt3a induced higher CMI responses to gt1b and gt3a NS5B proteins than a DNA vaccine encoding a global consensus sequence (49), while a multiantigenic DNA vaccine cocktail encoding gt1b and gt3a NS3, NS4, and NS5B proteins significantly improved the responses to NS3 and NS5B compared to those induced by the individual-genotype vaccines (49). Further experiments should be conducted to assess whether a DNA cocktail vaccine comprising the envelope constructs reported in this study in combination with the DNA vaccine encoding NS proteins can elicit both anti-E1E2 NAb and CMI responses to the NS proteins. This strategy will ensure that the induced antibodies will either prevent HCV infection of host cells or reduce the number of HCV-infected hepatocytes that will in turn be eliminated by the CD8^+^ T cell response. In fact, the induction of a broad CMI may be seminal to protect against infection with heterologous HCV strains (110, 111).

In summary, these results have significant implications for the design and development of a HCV prophylactic vaccine and demonstrate the flexibility and unique ability of the sE1/sE2-IMX313P strategy to elicit HCV-specific immune responses after homologous and heterologous prime-boost immunization. These results also support further development and testing of these vaccine constructs in larger animals, most likely in combination with DNA vaccines which elicit CMI.

## Ethics Statement

Six to eight weeks old female Balb/C mice were obtained from the University of Adelaide Laboratory Animal Services. Mice were housed in HEPA-filtered individually vented cages in The Queen Elizabeth Hospital animal house and were used in accordance with the SA Pathology and University of Adelaide animal ethics committee guidelines.

## Author Contributions

MM designed and performed almost all the experiments and wrote the manuscript. DW helped in data analysis and reviewing the manuscript. JT, DC, and LE-S performed the HCV neutralization assays and reviewed the manuscript. AU and RB performed the HCVpp neutralization assays. BG-B and EG conceived the study, helped in data analysis, and reviewing the manuscript.

### Conflict of Interest Statement

The authors declare that the research was conducted in the absence of any commercial or financial relationships that could be construed as a potential conflict of interest.

## Supplementary Material

Supplementary Figure S1 Expression of E1 and E2 by immunofluorescence analysis. Fluorescence micrograph of HEK293T cells transfected with the different DNA vaccine constructs. Cells were fixed and processed 48 h post-transfection. Protein expression was analyzed with anti-E1 (MyBiosource) and anti-E2 (Virostat) antibodies. Nuclei were stained with Hoechst 33342. Cells transfected with pVax served as controls for non-specific fluorescence. Scale bar = 50 μm.
